# Animal Models of Undernutrition and Enteropathy as Tools for Assessment of Nutritional Intervention

**DOI:** 10.3390/nu11092233

**Published:** 2019-09-16

**Authors:** Emmeline Salameh, Fanny B. Morel, Mamane Zeilani, Pierre Déchelotte, Rachel Marion-Letellier

**Affiliations:** 1 UniRouen, Inserm UMR 1073 Nutrition, Inflammation and Gut-Brain Axis, Normandie University, 76183 Rouen, France; Emmeline.salameh@etu.univ-rouen.fr (E.S.); Pierre.dechelotte@chu-rouen.fr (P.D.); 2 Nutriset SAS, 76770 Malaunay, France; Fmorel@nutriset.fr (F.B.M.); Mzeilani@nutriset.fr (M.Z.); 3 Department of Nutrition, Rouen University Hospital, 76183 Rouen, France

**Keywords:** undernutrition, environmental enteric dysfunction, enteropathy, gut barrier

## Abstract

Undernutrition is a major public health problem leading to 1 in 5 of all deaths in children under 5 years. Undernutrition leads to growth stunting and/or wasting and is often associated with environmental enteric dysfunction (EED). EED mechanisms leading to growth failure include intestinal hyperpermeability, villus blunting, malabsorption and gut inflammation. As non-invasive methods for investigating gut function in undernourished children are limited, pre-clinical models are relevant to elucidating the pathophysiological processes involved in undernutrition and EED, and to identifying novel therapeutic strategies. In many published models, undernutrition was induced using protein or micronutrient deficient diets, but these experimental models were not associated with EED. Enteropathy models mainly used gastrointestinal injury triggers. These models are presented in this review. We found only a few studies investigating the combination of undernutrition and enteropathy. This highlights the need for further developments to establish an experimental model reproducing the impact of undernutrition and enteropathy on growth, intestinal hyperpermeability and inflammation, that could be suitable for preclinical evaluation of innovative therapeutic intervention.

## 1. Introduction

Acute malnutrition is a major public health problem in low-income countries, affecting 51 million children under the age of five [1]. Severe acute malnutrition (SAM) in children increases the risk of impaired cognitive development, later chronic diseases, morbidity and mortality (12.6% of children under 5 years) [2,3]. Undernutrition is also often associated with environmental enteropathy (EE), also known as environmental enteric dysfunction (EED) [4]. Indeed, an observational study in Zambia showed that EED in SAM children with diarrhea was characterized by villus blunting, suggesting a lower nutrient absorptive area in the small intestine [5]. Later, the same investigator reported higher leucocyte infiltration in addition to villus blunting in this condition [6]. Intestinal permeability, assessed by lactulose:mannitol ratio, was also inversely correlated to villus height [6]. This data suggests that SAM children may develop EED and therefore EED may negatively affect nutrients absorption. EED has also been associated with an altered therapeutic response and fecal calprotectin, a sign of inflammation, and has been related with mortality [7]. These triumvirate, i.e., chronic intestinal inflammation, hyperpermeability and villous blunting, lead to reduced nutrient uptake, which affects growth and child development [4,8] and finally, enters into a vicious circle of worsening undernutrition state.

As studying gut barrier function in undernourished children is challenging, preclinical models have been developed to enable a better understanding of the mechanisms behind undernutrition and/or enteropathy. These models were also intended for the evaluation of novel therapeutics in undernutrition and/or EED. In this narrative review, we focus on available preclinical models of undernutrition and their effects on growth and gut barrier function. The respective advantages and limits of these models are discussed. A literature search using PubMed from inception to January 2019 was performed to identify relevant studies on experimental models of undernutrition and/or enteropathy. Manuscripts published in English were selected and reviewed.

## 2. Undernutrition Models

Undernutrition models are mainly induced by deficient diets, but some studies described genetic models of undernutrition. AMPK is involved in energy regulation and its genetic deletion has been investigated. For example, AMPK deficient *C. elegans* exposed to starvation in an early larval stage exhibited a developmental defect [9]. In mice, AMPK liver-specific deletion induced a lower body weight compared to wild-type mice fed with a low-protein and high-carbohydrate diet for 3 weeks [10]. These experimental genetic models are less relevant to human conditions, because acute malnutrition is mainly associated with dietary deficiencies. In the following sections, we focus on diet-induced undernutrition.

### 2.1. Caloric Restriction

Limitation of food provision in caloric restriction models varies from 15% to 50% in the literature (Table 1). Caloric restriction leads to undernutrition and has a full impact on body homeostasis. It is also associated with an impaired immune function as seen in undernourished children [11]. Twenty-four days of 25% caloric restriction induced weight loss was associated with thymus atrophy in BALB/C weanling mice, as a consequence of increased thymus cell apoptosis [12]. Altered cytokines production has been reported in numerous caloric restriction models: for example, a 30% caloric restriction for 14 days decreased systemic TNFα levels [13]. Similarly, prolonged moderate caloric restriction (−15%) induced lower cytokine mRNA levels of monocyte-chimioattractant protein 1 (MCP-1) in the liver and adipose tissue [14]. A lower MCP-1 level may be associated with lower accumulation of triglycerides in the liver [15]. No impact of caloric restriction on intestinal permeability has, however, been described [15].

In addition, caloric restriction might be stressful for rodents. Accordingly, in juvenile rats fed with 25% caloric restriction, plasma corticosterone levels were significantly increased [16]. Behavioral tests, such as forced swimming tests or O-maze, have been performed in rats with a 50% caloric restriction for 32 days, and these mice exhibited increased anxiety and depression symptoms [17].

### 2.2. Protein Undernutrition

#### 2.2.1. Maternal Protein Undernutrition

In the first 1000 days of life, including gestation and the first two years of life, children are at risk of developing short- and long-lasting consequences for their health, including undernutrition and impaired cognitive development. Indeed, a case-control study associated mother undernutrition with an increased risk of SAM in the children [40]. Pre-clinical models of fetal undernutrition have been developed and several effects on gut barrier in the offspring have been reported (Table 1). For instance, in female offspring from undernourished pregnant rats (8% protein diet), colonic tight junction Zonula Occludens 1 mRNA expression was decreased, without impacting on permeability to lipopolysaccharides (LPS), in comparison to offspring of pregnant rats receiving the standard 20% diet [18]. Another study with the same level of maternal protein restriction reported an increased ex vivo colonic hyperpermeability to small molecules in 35 days aged offspring [19]. In offspring from undernourished pregnant rats (receiving a 6% protein diet), the disaccharidase activity (lactase and sucrase) was increased in different segments of the intestine [41]. This may be an adaptive process in response to maternal undernutrition, in order to ensure a better absorption of carbohydrates, with the risk of a later stage development of obesity as a result of fetal programming [41]. In another study with piglets, maternal restriction (7.3% of protein) during gestation induced lower villus:crypt ratio [20]. Lower ileal [21] and duodenal [22] villus length has also been observed in low birth weight in piglets. This reduced villus height, and consequently absorptive area, may contribute to offspring undernutrition. Low inflammation has been observed in models of maternal undernutrition. Goat maternal protein restriction (60% of control diet) or energy restriction (60% of control diet) for example decreased plasma C3, C4, IgG, and IgM concentrations and jejunal IL-2 and IL-6 mRNA expression in offspring; this altered immune response may make the offspring more prone to bacterial infection [23]. In another study in sows, low energy diet (3.00 MCal DE/kg vs. 3.40 MCal DE/kg for control diet) was associated with higher ileal mRNA expression of IL-6 and TNF-α in offspring [20]. Thus, the effect of protein restriction on intestinal immunity may differ in the proximal and distal intestine, but this must be confirmed in the same species. Protein–energy intrauterine undernutrition also induced anxiety-like behaviors and cognitive impairment in the offspring [42,43,44,45,46,47,48] which could contribute to altered feeding behaviors and to the vicious circle of undernutrition.

#### 2.2.2. Protein-Deficient Diet

As caloric restriction leads to stress, ad libitum deficient diets may be a better alternative in order to respect the ethics for animal research. Protein-deficient diets reflect nutritional deficiencies commonly observed in undernourished children and are commonly used in the literature. While low-protein diet is isocaloric to the standard diet, several studies demonstrated increased food intake in rodents at different protein proportions, despite weight loss [49,50,51]. Protein-deficient diets lead to undernutrition in rodents [25,26] and are associated with gut barrier dysfunction in some conditions (Table 1). A drastic model developed by Belmonte et al. showed that a protein-free diet impacts gut barrier function by reducing jejunal villus height, leading to a decreased nutrient absorption area [24]. By contrast, in weanling mice, a moderate to low-protein (LP) diet (7% of protein) for three weeks did not have this effect [25], signifying the impact of protein proportion on the severity of gut barrier architecture. In the same study, this diet further induced in vivo gut hyperpermeability that might be explained by jejunal tight junction protein modulation as decreased ZO-1 and increased claudin-2 mRNA levels [25]. Studies in undernourished rats fed with a more drastic low-protein diet (4%) for three weeks encountered no gut hyperpermeability to large molecules, but it did decrease colonic and ileal transepithelial electrical resistance, suggesting a higher intestinal permeability to small molecules [26]. This intestinal hyperpermeability might be explained by a decreased colonic and ileal occludin protein expression [26]. The effect of protein-deficient diets on inflammatory response is, however, controversial. First, Belmonte et al. did not observe any difference in plasmatic level of α-1-acid glycoprotein, a major acute phase protein among control and protein-free diet fed rats [24]. Similarly, a 3-week LP diet (7%) decreased MCP-1 jejunal release in stool [25]. By contrast, a higher inflammatory response with a 2-week LP diet has also been described. Indeed, the LP diet (2% of protein) induced high fecal myeloperoxidase (MPO) and lipocalin 2 (LCN-2) production in weaned mice [29]. A 4-week LP diet at 4% increased mRNA expression of cytokines such as TNF-α, MCP-1 and IL1-α in the liver [27]. LP-induced inflammation might be explained by higher susceptibility of undernourished mice to infection. Actually, a drastic weaned mice model of undernutrition (0.7% protein for 16 days) increased the number of monocytes and macrophages in bone marrow and blood, as compared to normonourished mice, thereby suggesting contribution of the LP diet in increasing susceptibility to infectious diseases [31]. In gnotobiotic piglets transplanted with human infant fecal microbiota, a low-protein diet (7.5%) induced lower oral human rotavirus vaccine efficacy characterized by decreased serum TNF-α, IFN-α, IFN-γ and IL-12 responses [52]. The same conclusion has been proposed in undernourished mice fed with an LP diet (2%) for six weeks after *Salmonella* and cholera vaccines [53]. By contrast, a 1-week LP diet (2%) in mice decreased leucocyte, peripheral lymphocyte, monocyte and polynuclear cells levels without inflammation differences, as compared to normonourished mice [30]. Fock et al. also observed leucopenia, with higher systemic IL-10 production in 2–3 months aged mice fed with a higher proportion of protein (4%) for 14 days [28]. Moreover, in undernourished mice from the same study, cultured cells from bone marrow, spleen and peritoneum with LPS in vitro, showed lower IL-6, TNF-α and IL1-β production [28]. In addition, a lower expression of macrophage CD-4 and TLR-4/MD-2 has been observed in this group, which could interfere with immune response to pathogens [28]. Lower TLR-4 expression might explain the lower mRNA expression of TNF-α by macrophage from malnourished mice fed with LP diet (2%) and the lower NF-κB activation after LPS challenge in vitro [54].

### 2.3. Regional Diets

In the LP diets previously described, mineral and vitamin proportions were adapted for rodents. In clinical settings, reduced micronutrients intake, such as vitamins and minerals, is also reduced, in addition to the low macronutrients intake in an undernourished child [55]. For this reason, specific animal diets have been formulated to mimic multiple nutrient deficiencies and to reflect local dietary patterns. Three regional diets have been used to induce undernutrition: the regional basic diet from Brazil, the maize diet and the Malawian diet (Table 1).

Regional Basic Diet (RBD) is an experimental rodent diet mimicking dietary pattern from the northeast Brazilian population, characterized by reduced protein (7%), fat (8.2%), and vitamins and minerals content, triggering clinical symptoms such as reduced weight gain in rats [56,57], which has been confirmed in later studies [32,33,58]. Three weeks of this RBD in weaned mice induced jejunal hyperpermeability [32], which was related to a decreased jejunal expression of the tight junction protein claudin-3 [32]. In addition, ileal permeability was also increased, despite the increased ileal claudin-2 and occludin mRNA expression [33]. In contrast, after 10 days of RBD in weaned mice, ileal occludin expression was decreased without measurable impact on gut permeability [59] and jejunal villous and crypt atrophy were noted [32]. In another series in mice, a 1-week RBD diet induced ileal crypt atrophy without changes in villus length, while 10 days of RBD decreased the villous:crypt ratio [33,59].

An article published in *The Lancet* in 1933 and redistributed by The Bulletin of the WHO in 2003 stipulated that the origin of Kwashiorkor syndrome, characterized by bilateral edema in undernourished children, was the maize diet in Ghana, a corn-based diet [60]. The maize diet is a diet high in carbohydrate, low in protein (6.4%), fat (3.4%) and minerals [35,61]. The maize diet was developed and first studied in monkeys to induce undernutrition [62]. In this study, Kwashiorkor-like syndrome was induced in monkeys fed for 41 weeks with maize diet, which included such symptoms as growth failure, weight loss, hepatic steatosis and bilateral edema [62]. The maize diet has also been administered in rodents and pigs, resulting in weight loss, hepatic steatosis and shorter villi and crypts in the intestine [35,36].

The Malawian diet (M8) is a prototypic diet based on the typical Malawian diet providing eight ingredients: corn flower, roasted peanuts, red kidney beans, pumpkin, bananas, onion tomatoes, mustard grains and water [34]. This diet has been shown to not cover the daily nutritional needs of gnotobiotic mice, leading to lean body mass loss and altered bone morphology, with liver, muscles and brain altered metabolism. However, intestinal barrier function or inflammation was not examined in this study [34].

### 2.4. Zinc-Deficient Diets

Prevalence of zinc deficiency is very high in young children and infants in low-income countries [63]. A study has shown that protein–energy malnutrition decreased zinc small intestinal absorption in rats [64,65]. In a mice model, low zinc intake (30 μmol/kg) for 28 days led to weight loss [66] while low zinc intake over 14 days did not affect weight [29]. Similarly, two weeks of a zinc-deficient diet did not alter intestinal villus:crypt ratio in mice [37]. Ileal permeability or plasma endotoxin concentration were not increased after eight weeks of a zinc-deficient diet [67]. Interestingly, a lower villus:crypt ratio was reported when mice under a zinc-deficient diet were challenged with an entero-adhesive *E. coli* (EAEC), which suggests a synergistic effect of infection and zinc deficiency in the development of enteropathy [37]. Two weeks of a zinc-deficient or zinc-free diet in mice did not alter inflammatory markers, such as fecal LCN-2 and MPO [29,68]. In *Shigella*-infected mice, a zinc-free diet prolonged *Shigella* gut colonization [68], while zinc supplementation (150 mg/L in drinking water) decreased *Shigella*-induced inflammation and enhanced weight recovery [68]. A zinc-deficient diet also decreased TNF-α, IL-1β and IL-6 production and ileal neutrophil infiltration, which might explain the increased susceptibility to EAEC [37]. Zinc deficiency led to an altered intestinal immune response to parasitic nematodes infection in mice [38]. Mechanisms behind an altered immune response involved lower production of IL-14, decreased levels of IgE, IgG1, lower eosinophils and impaired antigen-presenting cells function, which enhanced parasite survival and infectivity [38].

Taking all this together, and despite the varying levels of zinc depletion among the studies, these observations suggest an impaired immune response in mice fed with zinc-deficient diets, which leads to an increased vulnerability to pathogen infection and increased virulence (Table 1).

## 3. Enteropathy Models

Animal models of enteropathy have provided a wealth of information about gut barrier function, using microbial challenges or other gastrointestinal injury.

### 3.1. Microbiota Transplantation

Dysbiosis associated with child undernutrition varies from country to country [69], but is often reported. For example, higher *Bacteroidetes* and lower *Firmicutes* enrichment and unusual abundance of *Prevotella* and *Xylanibacter* genus were reported in children from rural Burkina Faso, in comparison to European children [69]. A Bangladeshi study reported microbiota immaturity in undernourished children and more especially in SAM children [70]. Investigators from the AFRIBIOTA consortium have described, in growth stunted children, a decompartmentalization of microbiota composition along the gastrointestinal tract illustrated by orophyngeal taxa overgrowth and a higher prevalence of *Escherichia coli/Shigella* sp. and *Campylobacter* sp. [71]. Alteration of gut microbiota has also been described in experimental models of undernutrition, but discrepancies between species have to be underlined (Table 1). A three-week low-protein diet in mice induced higher relative abundance of Gram negative bacteria such as *Bacteroidetes* and *Enterobacteriaceae,* in the small intestine [25]; in contrast, the RBD described above was associated with a higher proportion of *Firmicutes* [72].

Gnotobiotic rodents have been used as models. Indeed, transplantation of bacterial strains targeted by IgA (IgA+ bacteria consortium) from undernourished children fecal microbiota induced small intestinal and colonic epithelial disruption into gnotobiotic mice fed with Malawi-8 diet [39]. Transplantation also triggered sepsis characterized by an increased cytokines production, such as IL-10, IL-12p40, IL-1β, IL-6 and MCP-1 [39]. Similarly, microbiota transfer from Kwashiorkor children into gnotobiotic mice fed with the Malawian diet induced weight loss, and metabolic perturbation in amino acid, carbohydrate and intermediary metabolism [73].

### 3.2. Pathogen-Induced Enteropathy

Child exposure to environmental bacteria could play a role in the development of EED, although it has not been fully demonstrated. One mechanism may be a shift in microbe-host interactions in the intestine, that might induce chronic inflammation frequently associated with undernutrition [4,74,75]. Models of EED have been developed with different types of pathogen infection, using either protozoans (*C. parvum* or *G. Lamblia*), or bacteria.

*Cryptosporidium parvum (C. parvum),* a parasitic invader of small intestine epithelial cells [76], is a major cause of diarrhea and gastroenteritis in children and adults worldwide [77,78,79]. *C. parvum* infection has a major impact on pediatric health in resource-limited countries because it increases morbidity and mortality in children [80]. A combination of undernutrition with repeated or prolonged *C. parvum* infection might be responsible for long-lasting consequences on physical and cognitive development in children [81,82]. *C. parvum* infection also contributes to the vicious circle of undernutrition and infection by inducing nutrients malabsorption [83]. A model combining *C. parvum* infection and undernutrition in neonatal mice has been described [84]. In baby mice, undernutrition was induced by maternal separation, limiting lactation time (4 h D4 *post-partum*, 8 h on D5 and 16 h from D6 to D14) [84]. *C. parvum* infection on D6 induced weight loss, with an additional effect in previously undernourished babies [84]. *C. parvum* infected-undernourished babies also displayed ileal villous atrophy and crypt hypertrophy and developed higher ileal inflammation, reflected by the increased TNF-α and IFN-γ tissue levels [84]. The same team later developed another model in weaned mice, combining a low (2%) protein diet with *C. parvum* oocysts infection [85]. *C parvum* infection in undernourished mice increased CD8 + CD103 + T cells and B cells lamina propria infiltration [59]. As previously reported, *C. parvum* infection exacerbated weight loss and led to decreased villous height and increased crypt depth [84,85]. A more recent study, combining *C. parvum* infection with an LP diet (7%) in mice, for three weeks, also reported impaired gut barrier with a lower villi:crypt ratio and a lower ileal occludin protein expression, while other tight junction proteins were not affected [59]. Two weeks of low-protein diet (2%) in *C. parvum*-infected mice also induced intestinal inflammation, with higher fecal LCN-2 and higher MPO activity [86].

*Giardia* is a non-invasive enteropathogen affecting the proximal small intestine and leading to acute watery diarrhea [87,88,89,90]. Thus, its potential implication in the occurrence of enteropathy has been investigated. *Giardia* infection led to a disrupted villus architecture and was associated with a higher lactulose:mannitol ratio, indicating increased intestinal permeability [91]. These features may contribute to nutrient uptake deficiency and gut barrier dysfunction [91,92,93]. A murine model combining a protein deficient (3%) diet with *Giardia Lamblia* infection demonstrated decreased nutrient absorption [94]. Undernourished weaning mice infected with *Giardia muris* developed small intestinal injury, villus atrophy and brush border duodenal and jejunal enzymatic depletion [95]. *Giardia lamblia* cultures from symptomatic and asymptomatic children inoculated in axenic Gerbils (*Meriones unguiculatus*) both induced growth retardation, suggesting that silent enteric dysfunction caused by the pathogen could already impair growth [96]. Besides its effects on weight loss, *Giardia* infection also induced gut barrier dysfunction [97,98]. At D11 post infection, severely damaged villi, increased lymphonuclear cells lamina propria infiltration and severe ileitis were observed [98]. In weaned undernourished gerbils, persistent *Giardia lambia* infection induced crypt hyperplasia without villus blunting associated with eosinophils infiltration of villus and crypt units [99]. Yet, the pertinence of using *Giardia* as an EED trigger has been debated. Observational studies in Malawi suggested links between mortality and systemic and intestinal infection in SAM children with diarrhea, but did not find an association between the intestinal presence of *Giardia* and mortality or diarrhea evolution [7]. Similarly, investigators of the “Malnutrition and Enteric Disease” (MAL-ED) network observed that the number of *Giardia* pathogens was higher in non-diarrheal stools [100], while a meta-analysis of 12 longitudinal acute diarrhea studies did not find an association with *Giardia* [101]. Finally, *Giardia* infection was negatively correlated with linear growth during the first two years in the MAL-ED studies; in contrast, fecal MPO or neopterin were not increased in non-diarrheal stools [102,103].

**Figure 1:**
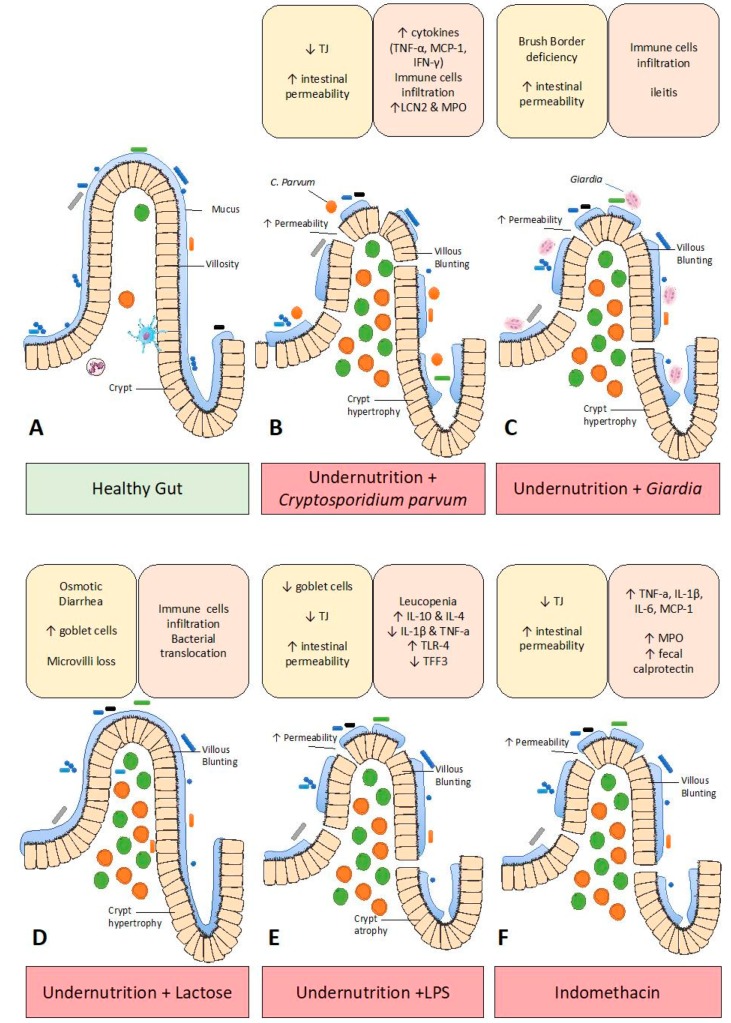
Experimental models of undernutrition and/or enteropathy and their respective impact on gut barrier function (yellow box) and inflammation (orange box). (**A**) Healthy gut. (**B**) Combination of undernutrition with *Cryptosporidium parvum* infection induced higher intestinal permeability, lower tight junction protein levels, villous blunting, crypt hyperplasia and inflammation. (**C**) *Giardia* infection induced intestinal hyperpermeability, villous blunting, crypt hyperplasia, ileal inflammation and immune cell infiltration. (**D**) Lactose-induced enteropathy induced villous blunting, increased goblet cells number, osmotic diarrhea, immune cell infiltration and bacterial translocation. (**E**) LPS-enteropathy in undernourished mice induced intestinal hyperpermeability and lower tight junction levels, fewer goblets. (**F**) Indomethacin-induced enteropathy led to intestinal hyperpermeability and intestinal inflammation.

Both *C. Parvum* and *Giardia* may thus induce some features of enteropathy in models of undernutrition, which are summarized in Figure 1.

In another model, described by Brown et al., a protein-deficient diet was combined with oral exposure to commensal *Bacteroidetes* and *E. coli* in mice. After induction, weight loss was associated with increased intestinal permeability and villus and crypt atrophy [25]. Additionally, ex vivo jejunal cytokine release (IL-6 and MCP-1) was increased [25]. Furthermore, acute inflammation induced by bacterial challenge may be too severe in comparison to the subclinical conditions observed during EED. Thus, non-infectious models might be a good alternative to induced controlled and reproducible inflammation and gut epithelial dysfunction.

### 3.3. Lactose-Induced Enteropathy

In undernourished children, mortality related to diarrhea is frequent [104]. In some undernourished children, secondary lactase deficiency may induce osmotic diarrhea as a consequence of lactose malabsorption [105]. Accordingly, lactose overload could be used to induce enteropathy in rodents (Figure 1). A high amount of lactose added to the diet for seven days induced diarrhea in rats associated with goblet cells hyperplasia and polymorphonuclear cell infiltration along the ileum, caecum and colon [106]. In undernourished rats with the RBD diet, provision of a saturated lactose solution (30 g/kg) over seven days led to osmotic diarrhea and weight loss [58,107]. Undernourished rats with diarrhea presented increased jejunal inflammation [58]. In the same study, higher bacterial translocation and a higher number of goblet cells were also indicative of gut barrier dysfunction [58]. Another study in rats fed with lactose reported a loss of colon microvilli and increased number of goblet cells [107]. In contrast, a modest (5%) lactose supplementation of the diet for four weeks did not alter protein and mucin content in intestinal mucosa or MUC2, MUC4 gene expression [108]. Of note, the use of lactose as an enteropathy trigger in preclinical models precludes the subsequent evaluation of therapeutic solutions containing high amounts of lactose. Finally, this kind of lactose-induced enteropathy is unlikely to occur in the clinical setting, which limits the clinical relevance of this model.

### 3.4. LPS-Induced Enteropathy

Lipopolysaccharides (LPS) are a lipid-based component of the outer membrane of Gram-negative bacteria, such as *Escherichia coli* or *Salmonella enterica* [109]. LPS induced inflammation after binding to CD14 and the Toll-like receptor, as well as the activation of transcription factors such as as NF-κB [110]. LPS has been used in undernourished animals (Figure 1). In weanling rats, undernutrition induced by a low-protein diet (5% of protein) for two weeks, with intraperitoneal injection of LPS (2 mg/kg) twice a week, resulted in impaired glucose clearance and insulin secretory response [111]. In another study, a low-protein diet (4%) for two weeks, in combination with intravenous LPS, induced leucopenia and severe depletion in bone marrow, spleen and peritoneal cavity cellularity 24 h later [28]. The LPS injection also triggered lower IL-1β levels in blood, spleen and bone marrow [112] and lower systemic TNF-α levels [30]. In a model of maternal undernutrition induced by 50% caloric restriction in pregnant rats, a single i.p. injection of LPS (500 μg/kg) worsened anorexia and weight loss in adult offspring 36 h later [113]. Moreover, LPS injection induced higher hypothalamic TNF-α levels in rats 6 h later suggesting hypothalamic inflammation [113]. Intravenous LPS increased plasmatic IL-6 levels in protein–energy malnourished rats [114].

LPS also alters gut morphology. Indeed, intraperitoneal injection of LPS at 5 to 30 mg/kg induced duodenal villus blunting [115,116]. Intraperitoneal LPS injection at 4 mg/kg reduced jejunal trefoil peptides and the number of goblet cells in mice [117]. A single subcutaneous injection of LPS (1 mg/kg) induced colonic hyperpermeability in rats 5 h after LPS challenge [118]. This early dysfunction of gut barrier function may result from tight junction protein dysregulation. Indeed, in vitro LPS at 50 μg/mL down-regulated occludin and ZO-1 expression and this effect was mediated by TLR-4 pathway [119,120,121]. LPS-induced gut barrier dysfunction was more severe in the ileum than in the colon day days later in rats [122]. LPS i.p injection (O55B7 10 mg/kg) increased colonic histologic score and induced global intestinal hyperpermeability 12 h later in mice [123]. In the latter study [123], LPS injection also decreased colonic ZO-1, occludin and claudin-1 protein expression.

Rodent models of endotoxemia have also been used to induce systemic inflammation. Accordingly, a single i.p injection of LPS (0.5 mg/kg–*E. coli* serotype 0111:B4) induced systemic inflammation 4 h after injection with higher IFN-γ, IL-6, MCP-1 and TNF-α levels [124]. Similarly, 10 h post LPS i.p. injection (*Escherichia coli* O26:B6–1.5 mg/kg), CD1 mice displayed increased cytokines levels (IL-1β, IL6, IL10, IL12, IFN-γ, TNF-α) [125]. In summary, although several studies using LPS injection have reported transient alterations of gut structure or function, the short duration of the effect and the variability of the response according to the dose and type of LPS make it difficult to use this model for the evaluation of therapeutic interventions targeting malnutrition and enteropathy.

### 3.5. Indomethacin-Induced Enteropathy

Non-steroidal anti-inflammatory drugs (NSAIDs) are among the most frequently used medications worldwide for routine relief of pain, fever, to manage various forms of arthritis or other inflammatory disorders, and to prevent or treat cancers [126,127]. As a drawback to their effectiveness, NSAIDs may damage the gastrointestinal (GI) tract and specifically, the small intestine [128,129,130]. As an inhibitor of cyclooxygenase (COX), NSAID, and more especially cyclooxygenase-2 (Cox2) inhibitor, will impair prostaglandins production such as PGE_2_ which is the most abundant in the small intestine and responsible for the regulation of mucus layer production [131]; accordingly, decreased PGE_2_ production will result in a decreased mucus layer thickness [131]. NSAID long term treatment will also impact microbiota composition with higher Gram negative bacteria abundance [128]. Clinical studies also indicated increased intestinal permeability in long-term NSAID-treated patients and intestinal inflammation reflected by increased fecal calprotectin concentration [132]. A decreased mucus layer and increased permeability will enhance mucosal exposure to luminal aggressive factors such as bile, enzymes and bacteria leading to exacerbated immune response [133]. Thus, NSAID-induced enteropathy, which is easily achieved with indomethacin in rodents, combines altered gut barrier function with intestinal inflammation (Figure 1). Subcutaneous injection of indomethacin (10 mg/kg) in mice resulted in an increased histopathological score in the small intestine at 12 and 24 h [134,135]. Intragastric indomethacin administration at 10 mg/kg increased the histopathological score 48 h later [136]. Indomethacin (10 mg/kg) subcutaneous injection in mice increased intestinal permeability and induced ulcers 24 h post administration [135]. These consequences have also been observed 24 h after oral indomethacin administration [137,138] in mice (10 mg/kg) and rats (15 mg/kg). Gut permeability increased over the first hours after a single indomethacin gavage in rats (20 mg/kg) and returned to normal four days post treatment [139]. Using a single administration may thus be inadequate for the pre-clinical evaluation of nutritional interventions over several days. Interestingly, a model of repeated gavages of indomethacin for six days (5 mg/kg per day) has been developed in mice and elicited a prolonged gut hyperpermeability [140]. Underlying mechanisms probably involved tight junction protein alterations, since lower claudin-1 and ZO-1 small intestine expression was observed 48 h after intragastric administration of indomethacin (10 mg/kg) in mice [136]. Indomethacin administration also led to epithelial barrier architecture disruption with a lower villus:crypt ratio, suggesting villus blunting, which is a marker of enteropathy [140]. In this model, gut inflammation is likely to result from the combination of mucosal injury and increased exposure to pathogens [133]. Indomethacin per os administration in rats (10 mg/kg) increased jejunal and ileal TNF-α ex vivo production 24 to 48 h after treatment [141]. Subcutaneous indomethacin injection (10 mg/kg) induced higher small intestine mRNA levels of TNFα, IL1-β and IL-6 12 h [134] and 24 h after injection [135]. Higher serum TNF-α cytokine level has been demonstrated in rats 12 h after subcutaneous injection (7.5 mg/kg) and lasted four days [142]. Single indomethacin gavage (10 mg/kg) induced higher TNF-α and MCP-1 mRNA levels into small intestine in rats 24 h later without TLR-4 protein expression modulation [143]. Indomethacin single gavage at 10 mg/kg in TLR-4-mutant mice did not induce small intestinal damages or TNF-α and MCP-1 mRNA expression modulation, suggesting that enteropathy development is independent of LPS/TLR-4/My-D88 pathway activation [143]. Moreover, indomethacin subcutaneous injection (10 mg/kg) induced iNOS activity 24 h later in rats [144]. Subcutaneous injection indomethacin (10 mg/kg) triggered jejunal–ileal iNOS activity and neutrophils activation preceded by TNF-α production ex vivo, thereby suggesting the NO role in intestinal injury induced by NSAID via TNF-α upregulation [141]. In a rat model of jejunoileitis, two indomethacin subcutaneous injections (7.5 mg/kg—24 h apart) induced higher small intestinal MPO activity four days after the first injection [142]. Indomethacin single gavage (20 mg/kg) increased fecal calprotectin, a marker of intestinal inflammation, from D5 to D7 after administration in rats [139]. Similarly, indomethacin gavage (5 mg/kg once a day for 6 days) in mice resulted in increased fecal calprotectin concentration [140]. In summary, indomethacin induces enteropathy in preclinical models characterized by higher histopathological score, intestinal inflammation and hyperpermeability. As previously reported for LPS challenge, indomethacin effect on gut barrier is still transient and protocols with repeated administrations are required to evaluate therapeutic strategies.

## 4. Conclusions

In the present review, we have reviewed and discussed the available animal models that may be useful to mimic the clinical features of SAM and EED. Understanding the physiological mechanisms involved during an episode of undernutrition associated with enteropathy is a critical step in developing novel therapeutic strategies. Human studies are obviously limited in terms of investigating gut function in undernourished children, in particular because of the lack of precise non-invasive methods. These limits do not apply to preclinical research. Animal models therefore offer an affordable tool to better investigate the mechanisms associated with undernutrition and enteropathy, and highlight potential biomarkers which can be translated back to the clinical condition.

Although undernutrition induced by micro and/or macronutrients deficiencies can impair growth, it is not sufficient to induce enteropathy. Several combinations of infection and undernutrition can induce growth failure and affect gut barrier, but these models seem to be specific of a pathogen and may lead to uncontrolled inflammatory response, which limit their validity. Development of non-infectious models of SAM with EED by associating undernutrition with triggers that induce growth failure with moderate and prolonged enteropathy appears to be the best approach so far to set up an easy and reproducible pre-clinical model allowing for the evaluation of therapeutic strategies.

## Figures and Tables

**Table 1:** Diet modifications and microbiota transfer: effects on growth and environmental enteric dysfunction (EED) development.

Preclinical Model	Growth	Gut Hyperpermeability	Gut Inflammation	Ref.
Caloric Restriction (CR)				
−15% CR	Weight loss	n/a	Lower MCP-1 mRNA (liver/adipose tissue)	[14]
−25% CR	Weight loss	n/a	Thymus atrophy	[12]
−30% CR	Weight loss	No gut hyperpermeability	Decreased systemic TNFα levels	[13]
Intrauterine Undernutrition				
8% of protein in gestational rats/20% in offspring	Low birth weight	Lower colonic ZO-1 mRNA expression	n/a	[18]
		No hyperpermeability to LPS		[18]
		Ex vivo colonic hyperpermeability to FSA		[19]
20% in gestating sows/low birth weight	Low birth weight	Lower villus length ileum/duodenum		[20,21,22]
7.3% in gestating sows/20% for piglets	Low birth weight	Lower villus:crypt ratio in piglets	Lower IL-6 and TNF-α mRNA in offspring (ileum)	[20]
Maternal protein restriction or energy restriction (60% vs. CT)	Low birth weight	n/a	Lower C3, C4, IgG, and IgM concentration in plasma offspring	[23]
			Decreased jejunal IL-2 and IL-6 mRNA expression in offspring	
Protein Energy Undernutrition				
0% of protein	Weight loss	Lower jejunal villus length	No difference in plasmatic α-1-Acid Glycoprotein	[24]
7% of protein	Weight loss	No villus atrophy/	Decreased MCP-1 macrophages release in vitro	[25]
		In vivo hyperpermeability		
		Lower jejunal ZO-1 and higher claudin-2 mRNA		
4% protein	Weight loss	No hyperpermeability to large molecules	n/a	[26]
		Hyperpermeability to small molecules	n/a	
		Lower colonic and ileal occludin	n/a	
		n/a	Higher TNF-α, MCP-1 and IL1-β production (liver)	[27]
			Leucopenia with higher systemic IL-10 production	[28]
			Lower CD-4 and TLR-4/MD-2 (macrophages)	
			Lower IL-6, TNF-a and IL1-B production by cultured cell from bone marrow, spleen and peritoneum after in vitro LPS treatment	
2% protein	Weight loss	n/a	Higher MPO and LCN-2 production	[29]
			Decreased leucocyte, peripheral lymphocyte, monocyte and polynuclear cells levels	[30]
			Lower TLR-4 expression	[28]
			Lower mRNA expression of TNF-α by macrophage in vitro	
			Lower NF-κB activation in vitro	
0.7% of protein	Weight loss	n/a	Increased monocytes and macrophages number in bone marrow and blood	[31]
			Higher monocyte arginase expression	
RBD	Weight loss	Jejunal hyperpermeability	n/a	[32]
		Decreased jejunal claudin-3 tight junction protein expression		
		Jejunal villous, crypt atrophy		
		Lower ileal basal short circuit current		[33]
		Higher ileal claudin-2 and occludin mRNA expression		
		Ileal crypt atrophy No villous length difference		
M8	Weight loss	n/a	n/a	[34]
MAIZE	Weight loss	Small mucosal atrophy/shorter villi and crypt	n/a	[35,36]
Zinc deficiency				
Zinc deficiency	Weight loss	No impact on villi/crypt ratio	No inflammation	[37]
		Decreased villi/crypt (EAEC -infected mice)	Decreased TNF-a, IL1-B and IL-6	
		No ileal hyperpermeability	Decreased ileal neutrophil infiltration	[38]
		No higher plasmatic endotoxin	Altered immune response to parasitic nematodes	
			Lower production of IL-4, Decreased level of IgE, IgG1	
			Lower eosinophils and impaired of antigen-presenting cells function	
Microbiota Transfer				
Cocktail of bacteria	Weight loss	Increased global intestinal permeability	Increased IL-6, MCP-1 (macrophages)	[25]
		Villus and crypt atrophy		
IgA+ bacteria consortium + M8 in gnotobiotic mice	Higher weight loss	Villus and crypt atrophy	Bacterial translocation	[39]
			Mucosal immune activation with neutrophils infiltration into lamina propria	
